# Dissecting the multifaceted contribution of the mitochondrial genome to autism spectrum disorder

**DOI:** 10.3389/fgene.2022.953762

**Published:** 2022-11-07

**Authors:** Leonardo Caporali, Claudio Fiorini, Flavia Palombo, Martina Romagnoli, Flavia Baccari, Corrado Zenesini, Paola Visconti, Annio Posar, Maria Cristina Scaduto, Danara Ormanbekova, Agatino Battaglia, Raffaella Tancredi, Cinzia Cameli, Marta Viggiano, Anna Olivieri, Antonio Torroni, Elena Maestrini, Magali Jane Rochat, Elena Bacchelli, Valerio Carelli, Alessandra Maresca

**Affiliations:** ^1^ IRCCS Istituto delle Scienze Neurologiche di Bologna, Programma di Neurogenetica, Bologna, Italy; ^2^ IRCCS Istituto delle Scienze Neurologiche di Bologna, UOSI Epidemiologia e Statistica, Bologna, Italy; ^3^ IRCCS Istituto delle Scienze Neurologiche di Bologna, UOSI Disturbi dello Spettro Autistico, Bologna, Italy; ^4^ Department of Biomedical and Neuromotor Sciences, University of Bologna, Bologna, Italy; ^5^ IRCCS Stella Maris Foundation, Department of Developmental Neuroscience, Pisa, Italy; ^6^ Department of Pharmacy and Biotechnology, University of Bologna, Bologna, Italy; ^7^ Department of Biology and Biotechnology “L. Spallanzani”, University of Pavia, Pavia, Italy; ^8^ IRCCS Istituto delle Scienze Neurologiche di Bologna, Programma Diagnostica Funzionale Neuroradiologica, Bologna, Italy

**Keywords:** mitochondrial DNA, mitochondrial haplogroups, universal heteroplasmy, autism spectrum disorder, autism risk

## Abstract

Autism spectrum disorder (ASD) is a clinically heterogeneous class of neurodevelopmental conditions with a strong, albeit complex, genetic basis. The genetic architecture of ASD includes different genetic models, from monogenic transmission at one end, to polygenic risk given by thousands of common variants with small effects at the other end. The mitochondrial DNA (mtDNA) was also proposed as a genetic modifier for ASD, mostly focusing on maternal mtDNA, since the paternal mitogenome is not transmitted to offspring. We extensively studied the potential contribution of mtDNA in ASD pathogenesis and risk through deep next generation sequencing and quantitative PCR in a cohort of 98 families. While the maternally-inherited mtDNA did not seem to predispose to ASD, neither for haplogroups nor for the presence of pathogenic mutations, an unexpected influence of paternal mtDNA, apparently centered on haplogroup U, came from the Italian families extrapolated from the test cohort (n = 74) when compared to the control population. However, this result was not replicated in an independent Italian cohort of 127 families and it is likely due to the elevated paternal age at time of conception. In addition, ASD probands showed a reduced mtDNA content when compared to their unaffected siblings. Multivariable regression analyses indicated that variants with 15%–5% heteroplasmy in probands are associated to a greater severity of ASD based on ADOS-2 criteria, whereas paternal super-haplogroups H and JT were associated with milder phenotypes. In conclusion, our results suggest that the mtDNA impacts on ASD, significantly modifying the phenotypic expression in the Italian population. The unexpected finding of protection induced by paternal mitogenome in term of severity may derive from a role of mtDNA in influencing the accumulation of nuclear *de novo* mutations or epigenetic alterations in fathers’ germinal cells, affecting the neurodevelopment in the offspring. This result remains preliminary and needs further confirmation in independent cohorts of larger size. If confirmed, it potentially opens a different perspective on how paternal non-inherited mtDNA may predispose or modulate other complex diseases.

## Introduction

Autism spectrum disorder (ASD) is a group of complex neurodevelopmental conditions with onset in childhood, characterized by highly heterogeneous clinical phenotypes. Neuropsychiatric manifestations consist of impairments in social communication and interaction, restricted and repetitive behaviors, often in association with attention deficit/hyperactivity, and intellectual disability, all present at different severity levels; additional co-morbidities may include epilepsy, sleep and gastrointestinal disorders (Masi et al., 2017). Based on a recent epidemiological survey on 8-year old children, the ASD prevalence is 18.5 per 1,000 in the United States (Maenner et al., 2020), and males are four times more frequently affected than females (Lyall et al., 2017).

Besides a putative environmental role, the clinical variability of ASD can be explained by the complex genetic landscape. Different genetic models have been described for ASD, including rare monogenic disorders, such as Fragile X syndrome, and with substantial contribution of rare variants of large effects including both single nucleotide variants (SNV) and copy number variation (CNV), either inherited or *de novo* (Turner et al., 2017; Ruzzo et al., 2019; Satterstrom et al., 2020; Toma, 2020). Furthermore, there is a polygenic contribution to the overall risk given by multiple common variants of small effect (Iakoucheva et al., 2019; Toma, 2020). Recently, the largest exome sequencing study identified more than 100 high confidence ASD susceptibility genes implicated in brain development and synaptic transmission (Satterstrom et al., 2020). The genetic background of rare exonic variants in ASD susceptibility genes can also modulate the penetrance of large-effect variants, as we recently demonstrated for *NRXN1* microdeletion carriers (Cameli et al., 2021)**.** In addition, the burden of non-coding mutations has been demonstrated to contribute to the cumulative risk for ASD (Zhou et al., 2019).

In this complex scenario, mitochondria may also have a role in the pathogenesis of ASD. Several studies have described a mitochondrial dysfunction in ASD patients and mitochondrial DNA (mtDNA) sequence variations have been investigated (Pei and Wallace, 2018; Rose et al., 2018; Citrigno et al., 2020; Pecorelli et al., 2020). Mitochondria are cytoplasmic organelles, sites of important metabolic processes and energetic hubs through the ATP production by oxidative phosphorylation (OXPHOS) (Spinelli and Haigis, 2018). Each cell contains a variable amount of mitochondria with hundreds to thousands copies of the small circular double stranded mtDNA, encoding for only 13 key proteins, all OXPHOS components, synthesized through a specific mitochondrial translation machinery, which includes 22 tRNAs and 2 rRNAs (Carelli and Chan, 2014). Mitochondrial genetics has peculiar rules, such as maternal inheritance, high mutational rate and, due to the multiple copies, a condition of heteroplasmy/polyplasmy, reflecting the co-existence of different sequence variants of the DNA molecules within the same cell/tissue (Carelli and Chan, 2014). Interestingly, a burden of low-level heteroplasmic variants (<2%) is inherited, including pathogenic mtDNA mutations that may clonally expand in specific cells or tissues, giving rise to clinical manifestations once a threshold of mutant load is overcome (Guo et al., 2013; Payne et al., 2013; Stewart and Chinnery, 2021).

While mtDNA pathogenic mutations, as well as mutations in the about 1,200 nuclear genes encoding mitochondrial proteins (Rath et al., 2021), may be causative for a large group of heterogeneous pathologies collectively defined as “mitochondrial diseases”, other mtDNA variants, some pathogenic and some with a possible milder functional impact, have been reported as predisposing to different neurological diseases, including ASD (Graf et al., 2000; Weissman et al., 2008; Avdjieva-Tzavella et al., 2012; Patowary et al., 2017; Pei and Wallace, 2018; Valiente-Pallejà et al., 2018; Varga et al., 2018). Similarly, mtDNA haplogroups, clusters of related haplotypes, and the mtDNA cellular content have been investigated in relation to ASD (Kent et al., 2008; Giulivi et al., 2010; Álvarez-Iglesias et al., 2011; Hadjixenofontos et al., 2013; Chen et al., 2015; Wang et al., 2016; Chalkia et al., 2017; Valiente-Pallejà et al., 2018; Singh et al., 2020), although with controversial results. Moreover, due to the mitogenome maternal inheritance, paternal mtDNA has been poorly investigated, with a few studies merging fathers with the independent controls (Hadjixenofontos et al., 2013; Chalkia et al., 2017). Recently, ASD behavioral endophenotypes have been described in a mouse model carrying a missense mutation in the *mt-Nd6* gene (Yardeni et al., 2021), which in humans is associated with a variable phenotype including the severe Leigh syndrome or a milder combination of optic neuropathy and cerebellar ataxia (Malfatti et al., 2007).

In the current study, we took advantage of a deeply phenotyped cohort of 98 families, with at least one individual affected by ASD, to analyze the possible contribution of mtDNA in the cumulative risk for ASD. We performed deep sequencing of the entire mitogenome and quantified mtDNA cellular content in the probands, as well as their parents and unaffected siblings, correlating these molecular findings to the clinical severity.

## Materials and methods

### ASD patients

This observational study was implemented in accordance with the STROBE (Strengthening and Reporting of Observational Studies in Epidemiology) guidelines (von Elm et al., 2008) and the ethical principles laid down in the Declaration of Helsinki. The study was approved by the Institutional Ethical Committee (“Comitato Etico di Area Vasta Emilia Centro-CE-AVEC”) (code CE 14060). All consecutive subjects referred to the Unit Autism Spectrum Disorders were informed and invited to participate to this study during their routine visits. Recruitment took place on a voluntary basis and all families gave informed written consent.

The study cohort consisted of 98 families (Supplementary Table S1), of which 73 simplex, with only one individual affected by ASD, 22 multiplex, with more than one affected, and three multiplex broad, with other family members showing neurodevelopmental manifestations, all recruited at the Unit Autism Spectrum Disorders of the IRCCS Institute of Neurological Sciences of Bologna (Bologna, Italy) between July 2016 and October 2019. A total of 369 individuals were included in the study, encompassing 117 with an ASD diagnosis (24 F/93 M), 193 parents (98 F/95 M) and 59 siblings (25 F/34 M). ASD was diagnosed according to the Diagnostic and Statistical Manual of Mental Disorders (fifth edition) (American Psychiatric Association, 2013) and confirmed by both Autism Diagnostic Observation Schedule-Second Edition (ADOS-2) (Lord et al., 2012) and Childhood Autism Rating Scale-Second Edition (Schopler et al., 2010).

For the mtDNA haplogroup distribution, an independent cohort of 127 Italian families, previously characterized (Bacchelli et al., 2020), was used as a replication sample for the present study. This cohort consisted of 128 individuals with ASD (106 M/22 F) and 238 parents (111 M/127 F). Both cohorts were compared to an in-house control group made of 242 Italian individuals (129 M/113 F).

### Deep sequencing of mtDNA

Total DNA was extracted from peripheral blood using the QIAamp DNA Blood Maxi Kit (Qiagen, Hilden, Germany), following the manufacturer’s instructions. Direct sequence analysis of the entire mitogenome was performed on total DNA by Next Generation Sequencing (NGS) (Caporali et al., 2018). Briefly, the mitogenome was amplified in two long-range PCR using a high-fidelity Taq DNA polymerase (PrimeSTAR Max DNA Polymerase, Takara). The NGS library was prepared by Nextera XT (Illumina Inc, San Diego, CA) and sequenced as 150-bp paired-end reads on NextSeq500 platform (Illumina Inc, San Diego, CA), using a High Output Kit (300 cycle). BCL files were demultiplexed and converted to the FASTQ format with the Illumina standalone bcl2fastq program (v2.20.0.422).

For mtDNA haplogroup affiliations and private variants analysis, Fastq files were analyzed with MToolBox v1.2 (Calabrese et al., 2014), considering over 15% heteroplasmic variants. This 15% threshold was chosen considering the minimum heteroplasmy level in blood cells associated with phenotypic expression of the MELAS mutation (Mancuso et al., 2014). Haplogroup affiliations are in agreement to the current nomenclature, mtDNA tree Build 17 on PhyloTree.org (Van Oven and Kayser, 2009). The H* haplogroup is an arbitrary group containing all H clades except from the otherwise specified clades. Private variants analysis focused on pathogenic potential of missense and tRNA variants: the Mitimpact 3D database was used for missense variants (Castellana et al., 2015), and MitoTip in the MITOMAP database (http://www.mitomap.org) for tRNA variants (Lott et al., 2013; Sonney et al., 2017). We considered as pathogenic the confirmed pathogenic variants in the MITOMAP database, and the rare variants never found in GenBank database and predicted deleterious or likely deleterious (CADD Phred score >20, MToolBox score >0.43, MitoTip raw score >12.66).

To analyze low-level heteroplasmy, Fastq files were analyzed with MToolBox v1.2 (Calabrese et al., 2014) and an in-house pipeline. Specifically, reads were aligned to the human reference genome (comprising the rCRS) using BWA (Li and Durbin, 2009). Aligned reads were treated for realignment with GATK (DePristo et al., 2011) and for duplicate removal with PicardTools (http://picartools.sourceforge.net). Reads uniquely mapped to the mtDNA reference genome and with a mapping quality score ≥20 were then extracted and used for variant calling with two different callers: the Unified Genotyper of GATK and the mutation-server DetermineVariants, a stand-alone of the mtDNA-server pipeline (Weissensteiner et al., 2016). In both callers, positions with a base quality score ≥20 were considered. Only mono-allelic sites were retained and calls were further filtered in order to avoid an excessive strand bias using a SOR (StrandOddsRatio) value ≤4, following the GATK Best Practice for variant filtration. Finally, a consensus of the variants called by the two callers and MtoolBox was created and universal heteroplasmy analysis was carried out on Single Nucleotide Variants. To check the contamination we used Haplocheck 1.3.3, a component of the Mitoverse platform (Weissensteiner et al., 2021). One sample (BEL_39.1), belonging to the “fathers” group, resulted contaminated, with contamination level >1% and “Used Heteroplasmies Minor” >0. This sample was included in the haplogroup analysis, since the contamination level (10.7%) did not affect the haplogroup assignment. On the contrary, we excluded this sample from the low-level heteroplasmy analysis, since the latter examined heteroplasmies <15%.

### Mitochondrial haplogroup definition from SNPs array data

We reanalyzed the genotyping data from Infinium Psych Array -24v1.1 (Illumina Inc, San Diego, CA) (Bacchelli et al., 2020), to define mitochondrial haplogroups in the Replica cohort, selecting these nucleotide markers: 2706A (H), 1438A (H2), 72C (HV0), 10034C (I), 16069T (J), 3010A (J1-H1), 7476T (J2), 11251G (JT), 10550G (K), 10688G (L2′3′4′6), 1018G (L3), 13650C (L3′4), 15043A (M), 9540T (N), 10238C (N1), 13780G (N1a), 12705C (R), 1888A (T), 11467G (U), 1811G (U2′3′4′7′8′9), 16270T (U5), 3197C (U5a′b), 3348G (U6). Samples belonging to haplogroup U5, selected for carrying the 11467G, 16270T and 3197C variants, were further investigated to discriminate sub-haplogroups U5a and U5b. The mtDNA positions 150C, 14793A and 16256C were genotyped by direct sequencing. Individuals carrier of the 14793A>G and 16256C>T nucleotide changes were defined as U5a, whereas individuals carrier of the 150C>T nucleotide change as U5b.

### MtDNA content assessment

MtDNA content/cell was assessed by Real Time-PCR, using a previously established method (Giordano et al., 2014). Briefly, a mitochondrial (*MT-ND2*) and a nuclear gene (*FASLG*) were co-amplified in a multiplex reaction and their concentration in each sample was determined by absolute quantification.

### Nuclear DNA *de novo* mutations

Whole-genome sequencing (WGS) was carried out in 91 out of 98 ASD families as part of the “New York Center for Collaborative Research in Common Disease Genomics” project, funded by the National Human Genome Research Institute (“NHGRI”) under Award Number 3UM1HG008901. Alignment and post-processing were performed as outlined by the Center for Common Disease Genomics project (https://github.com/CCDG/Pipeline-Standardization/blob/master/PipelineStandard.md). *De novo* variant identification was performed using family-level joint-genotyped VCF as input and FamSeq tool (Peng et al., 2013, 2014), as well as genotype calls generated by GATK’s HaplotypeCaller. We retained only high confidence *de novo* variant call supported by both FamSeq and GATK. High-confidence calls are *de novo* call that meets the following criteria: child’s/mother’s/father’s DP ≥ 12; child’s/mother’s/father’s GQ > 20; child’s AB = 0.30 ≤ child’s AB ≤ 0.70. We also filtered high confidence *de novo* calls further by excluding all sites that are present in GnomadGenomes and GnomadExomes combined population with a MAF <0.1% (https://gnomad.broadinstitute.org/) (Gudmundsson et al., 2022).

### Statistical analyses

Frequency distributions of mtDNA haplogroups and private variants were compared by Chi-square/Fisher’s exact test. *p*-values were adjusted by the Benjamini–Hochberg false-discovery rate (FDR) multiple testing correction (Benjamini and Yekutieli, 2001); after the correction a q-value < 0.10 was considered statistically significant.

One way ANOVA and Tukey’s multiple comparisons test were performed to evaluate differences in nuclear DNMs content and parents’ age at time of conception amongst different mtDNA haplogroups.

For mtDNA content, we first checked for normality of the data distributions with three different normality tests (D'Agostino & Pearson omnibus, Shapiro-Wilk and Kolmogorov-Smirnov). Next, the Mann Whitney test was used to compare siblings and ASD patients, whereas the Kruskal–Wallis test with Dunn’s multiple comparisons test was used to compare unaffected females, ASD females, unaffected males and ASD males. Two-sided *p*-values are presented.

Descriptive statistics were calculated for all variables and groups of interest, presenting continuous variables as means and standard deviations and categorical variables as absolute and relative frequencies. Fisher’s exact or chi-square tests and *t*-test or Wilcoxon-Mann-Whitney test were used to compare variables among groups of interest.

Two regression analyses were performed. In the first analysis the outcome was the presence or absence of ASD. For ASD risk evaluation, to eliminate the potential bias due to the higher number of ASD subjects compared to the unaffected siblings, we selected only families with at least one unaffected sibling. A conditional logistic regressions models (matched case-control analysis) (Pearce, 2016) was performed to adjust for the intra-familiar genetic background, excluding variables shared between siblings (i.e., mtDNA haplogroup). We evaluated the association between ASD condition and the following variables: 1) mtDNA content, 2) gender, subjects with variants at 3) 15%–5%, 4) 4.9%–1%, 5) 0.9%–0.5% and 6) 0.4%–0% heteroplasmy, 7) mtDNA and 8) nuclear DNMs (all subject-specific variables). Since the maternal and paternal ages at the time of conception were correlated to each other and the paternal age itself was highly correlated to the number of nuclear DNMs, we included only the latter in this analysis. The family was the cluster variable and likelihood ratio (LR) test was used to compare conditional models vs*.* classical logistic models.

In the second analysis the outcome was the ASD severity, classified according to the ADOS-2 criteria in “mild”, “moderate” and “severe”. Due to the small number of subjects classified as “mild”, we aggregated the categories “mild” and “moderate”. Univariable and multivariable generalized linear mixed models (GLMM) with binary response (Galbraith et al., 2010) were performed to take into account the family structure. We evaluated the association between ASD severity and the following variables: 1) maternal and 2) paternal age at time of conception, 3) paternal haplogroup (parents-specific variables), 4) mtDNA content, 5) gender, 6) family type (simplex or multiplex), 7) subject haplogroup, 8) the overall potential pathogenic mutations in mtDNA coding genes and those specifically in 9) CI, 10) CIII, 11) CIV and 12) CV, 13) the overall missense mutations in 14) CI, 15) CIII, 16) CIV and 17) CV, subjects with variants at 18) 15%–5%, 19) 4.9%–1%, 20) 0.9%–0.5% and 21) 0.4%–0% heteroplasmy, 22) mtDNA and 23) nuclear DNMs (all subject-specific variables). As in the previous analysis, family was the cluster variable and, considering the small sample size of several variable groups (Ali et al., 2019), LR test was performed to compare GLMM and standard logistic models.

In both regression analyses a number of preliminary models were therefore fitted, adding one by one all the eligible variables according to the *p*-value ascending order obtained with the univariable analyses. Following a stepwise approach only the variables with a *p*-value < 0.2 were selected and included in the final multivariable models. Results were presented as Odds Ratio (OR) and relative 95% Confidence Interval (95% CI), reporting the *R* ^2^ for each multivariable model. Statistical analyses were carried out using GraphPad Prism 6.0, Stata SE, version 14 (StataCorp, College Station, TX, United States) and IBM SPSS Statistics for Windows, version 20.0 (IBM Corp, Armonk, N.Y, United States) software.

## Results

### The impact of maternal and paternal mitochondrial haplogroups on the risk of ASD in offspring

MtDNA haplogroups impact mitochondrial physiology, acting as risk or protective factors for a variety of pathological conditions. Previous studies excluded a role of mtDNA variability on the susceptibility of ASD (Kent et al., 2008; Álvarez-Iglesias et al., 2011; Hadjixenofontos et al., 2013), whereas a more recent study described differences in mtDNA haplogroup distribution in ASD patients, as derived from gene array analysis (Chalkia et al., 2017).

In this study, we performed NGS of the complete mitogenome in all individuals of our test cohort (n = 369), specifically 117 ASD patients, 98 mothers, 95 fathers and 59 healthy siblings, defining mtDNA super-haplogroups, haplogroups, sub-haplogroups, and specific clades (Supplementary Table S1). Deep NGS has the advantage of avoiding the bias that might have been introduced by the techniques used in previous studies, i.e., re-analysis of array or whole exome sequencing. Specifically, the SNP-array technique analyzes only a limited number of known variants, not allowing the identification of private and rare variants, as well as accurate heteroplasmy quantification. On the other end, in the whole exome sequencing data, reads mapping on mtDNA are usually not sufficient to obtain uniform and deep coverage, thus affecting low heteroplasmy variant calling.

To evaluate haplogroup distributions in the ASD patients and their fathers, we extracted from our test cohort of 98 families those of Italian origin (77 paternal and 74 maternal lineages) and compared them with an available Italian cohort of 242 healthy individuals (Table 1). While the ASD maternal lines showed a frequency comparable with the control group, we found a significant increase of super-haplogroup UK, haplogroup U and sub-haplogroup U5a in the paternal lines compared to controls (Table 1).

**TABLE 1:** Mitochondrial haplogroups distribution in the Italian ASD paternal and maternal lines from the test cohort, compared to Italian healthy controls.

Super-haplogroups	Haplogroups	Sub-haplogroups	Paternal			Maternal			CTRLs	
			N	%	q-value	N	%	q-value	N	%
H			27	35.1	0.401	24	32.4	0.401	101	41.7
		H*	17	22.1	0.912	13	17.6	0.863	65	26.9
		H1	6	7.8	0.925	7	9.4	0.863	14	5.8
		H3	2	2.6	0.988	2	2.7	0.988	11	4.5
		H5	2	2.6	0.988	2	2.7	0.988	11	4.5
JT			10	13.0	0.401	14	18.9	0.745	51	21.1
	J		5	6.5	0.863	4	5.4	0.863	27	11.2
	T		5	6.5	0.915	10	13.5	0.864	24	9.9
UK			30	39.0	0.001*	17	23.0	0.401	42	17.4
	U		20	26.0	0.044*	14	18.9	0.536	27	11.2
		U*	2	2.6	0.925	1	1.3	1	3	1.2
		U1	2	2.6	0.925	2	2.7	0.863	3	1.2
		U2	3	3.9	0.863	1	1.4	1	3	1.2
		U3	4	5.2	0.863	5	6.8	0.863	5	2.1
		U4	0	0.0	0.863	3	4.1	0.864	5	2.1
		U5a	8	10.4	0.085*	1	1.4	1	4	1.7
		U5b	1	1.3	1	1	1.4	1	4	1.7
	K		10	13.0	0.536	3	4.1	1	15	6.2
Others			10	12.9	0.401	19	25.7	0.401	48	19.8
	HV		2	2.6	1	3	4.1	0.912	6	2.5
	HV0		3	3.9	0.988	4	5.4	0.915	8	3.3
	I		2	2.6	1	2	2.7	1	9	3.7
	L		0	0.0	1	2	2.7	0.863	2	0.8
	M		3	3.9	0.328	1	1.4	0.863	0	0.0
	N		0	0.0	0.925	0	0.0	0.925	4	1.6
	R		0	0.0	-	0	0.0	-	4	1.6
	R0a		0	0.0	1	2	2.7	0.863	2	0.8
	W		0	0.0	0.863	3	4.1	0.864	5	2.1
	X		0	0.0	0.863	2	2.7	1	8	3.3
Total			77			74			242	

Distribution of mtDNA, super-haplogroups, haplogroups and sub-haplogroups in the Italian ASD, paternal (n = 77) and maternal (n = 74) lines from the test cohort, compared to Italian healthy controls (n = 242). * indicates significant q-values (<0.10). H* contained all H clades, except H1, H3, H5; U* contained all U clades, except U1, U2, U3, U4, U5.

**FIGURE 1:**
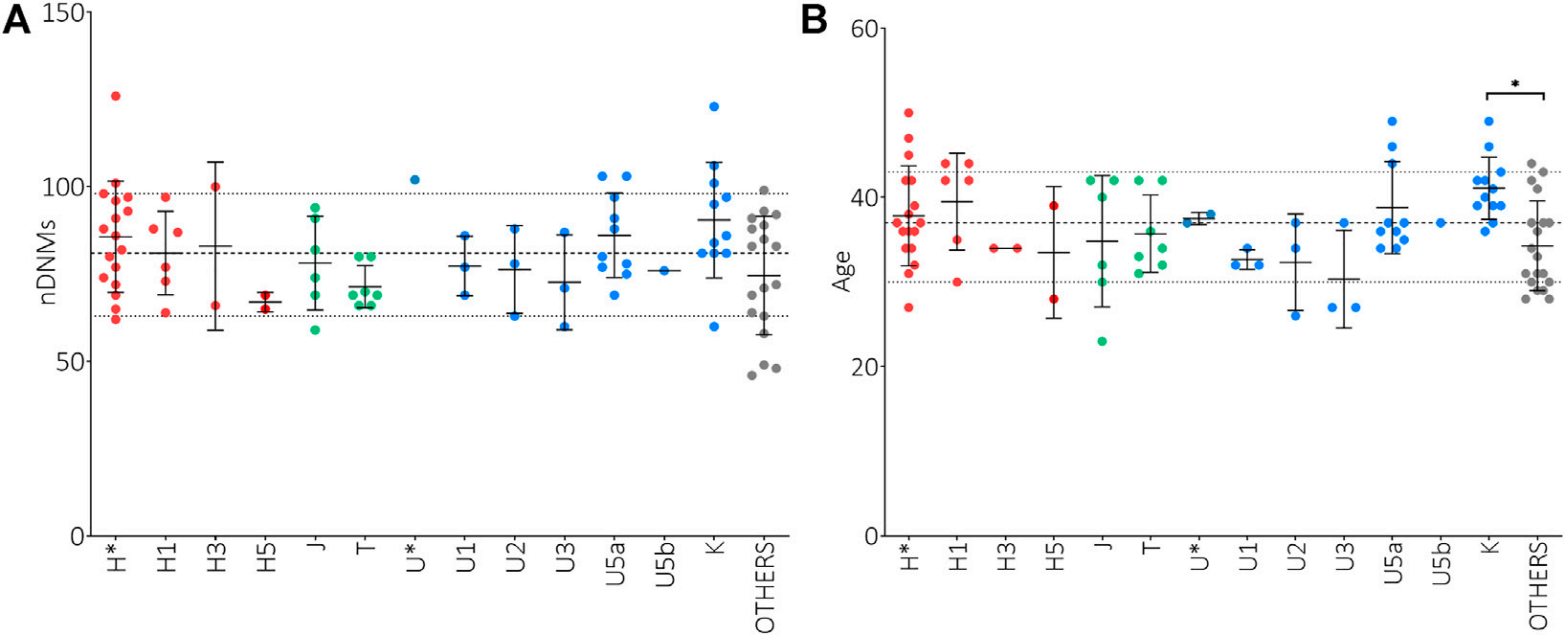
Analysis of nuclear *de novo* mutations (DNMs) in the offspring and paternal age at time of conception in relation to mtDNA haplogroups. **(A)** Number of nuclear DNMs in ASD probands stratified for paternal sub-haplogroups. **(B)** Paternal ages at time of conception stratified for sub-haplogroups. Ordinary One Way ANOVA test showed statistical significance only for the fathers’ age at time of conception (*p*-value = 0.0150) and the Tukey’s multiple comparisons test showed a significance only for K vs. “Others” comparisons (* *p*-value<0.05). Sub-haplogroups derived from the same root are reported in the same color. Dashed and dotted lines represent mean DNMs number and age ± standard deviations.

It has been reported that *de novo* mutations (DNMs) in the nuclear genome play a relevant role in the etiology of ASD (Michaelson et al., 2012; Neale et al., 2012; Iossifov et al., 2014; Jónsson et al., 2017; Turner et al., 2017; Breuss et al., 2020). To assess a possible correlation between the paternal mtDNA haplogroup and an increased risk to develop ASD in the offspring, we evaluated the number of nuclear DNMs in the ASD probands of our test cohort, as assessed by whole-genome sequencing, stratifying for sub-haplogroups. As shown in Figure 1A, the offspring of H*, U5a and K fathers had the highest number of DNMs, although without a statistical significance. Importantly, the number of DNMs in the germline, thus transmitted to the offspring, is strictly related to the paternal age at the time of conception (Goldmann et al., 2019). In our cohort, the fathers carrying sub-haplogroups H1, U5a and K were, on average, the oldest, but the only significant difference was between K and “Others” fathers’ age (Figure 1B). This may indicate that the association found in the test cohort between paternal haplogroups U5a and UK and ASD risk in the offspring could derive from the elevated age at time of conception of these fathers.

To replicate this association, we analyzed the haplogroups in an independent cohort of Italian ASD families (111 paternal and 127 maternal mtDNAs) (Bacchelli et al., 2020), previously analyzed by SNP-array (Table 2). In this independent cohort, we failed to highlight any significant difference in the haplogroup distributions among ASD maternal and paternal lines compared to the control group.

**TABLE 2:** Mitochondrial haplogroups distribution in Italian ASD paternal and maternal lines from an independent cohort, compared to Italian healthy controls.

Super-haplogroups	Haplogroups	Sub-haplogroups	Paternal			Maternal			CTRLs	
			N	%	q-value	N	%	q-value	N	%
H			40	36.0	0.574	43	33.9	0.574	101	41.7
		H*	33	29.7	0.705	28	22.1	0.139	87	36.0
		H1	7	6.3	1.000	15	11.8	0.302	14	5.8
JT			17	15.3	0.574	32	25.2	0.574	51	21.1
	J		4	3.6	0.146	17	13.4	0.861	27	11.1
	T		13	11.7	0.861	15	11.8	0.861	24	9.9
UK			30	27.0	0.367	22	17.3	1.000	42	17.4
	U		14	12.6	0.861	13	10.2	0.861	27	11.1
		U*	8	7.2	1.000	9	7.1	1.000	19	7.9
		U5a	4	3.6	0.705	2	1.6	1.000	4	1.7
		U5b	2	1.8	1.000	2	1.6	1.000	4	1.7
	K		16	14.4	0.146	9	7.1	0.861	15	6.2
Others			24	21.6	0.887	30	23.6	0.574	48	19.8
	HV0		6	5.4	0.825	4	3.1	1.000	8	3.3
	I		2	1.8	1.000	6	4.7	1.000	9	3.7
	L		3	2.7	0.582	4	3.2	0.582	2	0.8
	M		1	0.9	0.733	2	1.6	0.471	0	0.0
	N		3	2.7	1.000	9	7.1	0.139	4	1.7
	undetermined		9	8.1	1	5	3.9	0.243	25	10.3
Total			111			127			242	

Distribution of mtDNA, super-haplogroups, haplogroups and sub-haplogroups in Italian ASD, paternal (n = 111) and maternal (n = 127) lines from an independent cohort (Bacchelli et al., 2020), compared to Italian healthy controls (n = 242). H* contained all H clades, except H1; U* contained all U clades, except U5.

### The impact of mitochondrial private variants in the maternal and paternal lines on the ASD risk in offspring

Based on the availability of complete mtDNA sequences, we also analyzed private variants, i.e., those variants not marking the haplogroups (Supplementary Table S1). We focused this analysis on variants with a heteroplasmy level higher than 15%, checking also for their presence in other individuals along the same maternal line. We failed to detect any variant reported as *confirmed* pathogenic in the public database MITOMAP (http://www.mitomap.org/), neither in coding nor in tRNA genes, in the ASD probands, even in the heteroplasmic state. Furthermore, we also assessed the potential contributory effect of any missense variant, according to the pathogenicity predictions. We identified 94 variants, some occurring more than once: 60 on maternal lines and 55 on paternal mtDNAs (Supplementary Table S2). All these variants were already reported in the public database MITOMAP, except for the m.9238T>C/*MT-CO3*, m.7628C>T/*MT-CO2* and m.3820C>A/*MT-ND1* variants, which were all found in fathers, with only the m.7628C>T/*MT-CO2* change predicted as damaging. We also incidentally found the previously reported pathogenic mutation m.3890G>A/*MT-ND1* (Caporali et al., 2013) in the unaffected brother of the proband from Family 73; it was 18% heteroplasmic in blood, but it was not detected in other maternally related family members. This mutation has been associated with a clinical spectrum ranging from isolated optic atrophy to a severe neurological disorder characterized by progressive encephalomyopathy or Leigh syndrome. Other five variants were detected as heteroplasmic in the maternal lines, with two predicted as pathogenic (m.8884A>G/*MT-ATP6* and m.15884G>A/*MT-CYB*) both in Family 65. The mutational load of these two variants was highest in the proband (32% and 44%, respectively), when compared to the mother (1% and 7%) and the unaffected sister (6% and 1%). The m.15884G>A/*MT-CYB* is frequently found in the general population (0.8%) and is a mutational hot spot, as the G>C change is even more frequent (1.1%) (MITOMAP); on the contrary, the m.8884A>G/*MT-ATP6* was reported only once, apparently not associated with any disease, according to MITOMAP.

By analogy to missense variants, we also interrogated variants in tRNA genes. We found 29 variants, some occurring more than once, in 27 families, 22 on maternal and nine on paternal mtDNAs (Supplementary Table S3). All variants were reported in MITOMAP, only two predicted as pathogenic and three detected as heteroplasmic. The m.5628T>C in tRNA alanine predicted as *likely* pathogenic, was found homoplasmic in Family 18 and 97 times in GenBank; the m.7543A>G in tRNA aspartic acid, predicted as *possibly* pathogenic, was found homoplasmic in the two unrelated fathers from Families 30 and 66, and 47 times in GenBank. All heteroplasmic variants were predicted as *likely* benign. The m.5528T>C in tRNA tryptophan was found in Family 93 and its mutational load was 42% in the mother and 59% and 48%, respectively, in the two monozygotic twins, both with ASD. The m.7490A>G in tRNA serine (UCN) was found in Family 28, in the mother at 24% and in the unaffected sister at 21%, but it was undetectable in the ASD proband. The m.5939C>T in tRNA threonine was found at 87% heteroplasmy in the father of Family 55. Overall, none of these mtDNA variants was pathogenic *per se*, but a contributory role to the multifactorial pathogenesis of ASD remains a potential scenario.

To assess a possible predisposition effect, we evaluated the distribution of missense variants in the genes coding for OXPHOS complexes comparing the Italian families (maternal lines) with controls (Supplementary Table S4), failing to observe any significant difference. We also evaluated the distribution of private variants in tRNA genes, but the sample size was too small to identify any association (data not shown).

### Analysis of mtDNA low-level heteroplasmic variants and mtDNA content

**FIGURE 2:**
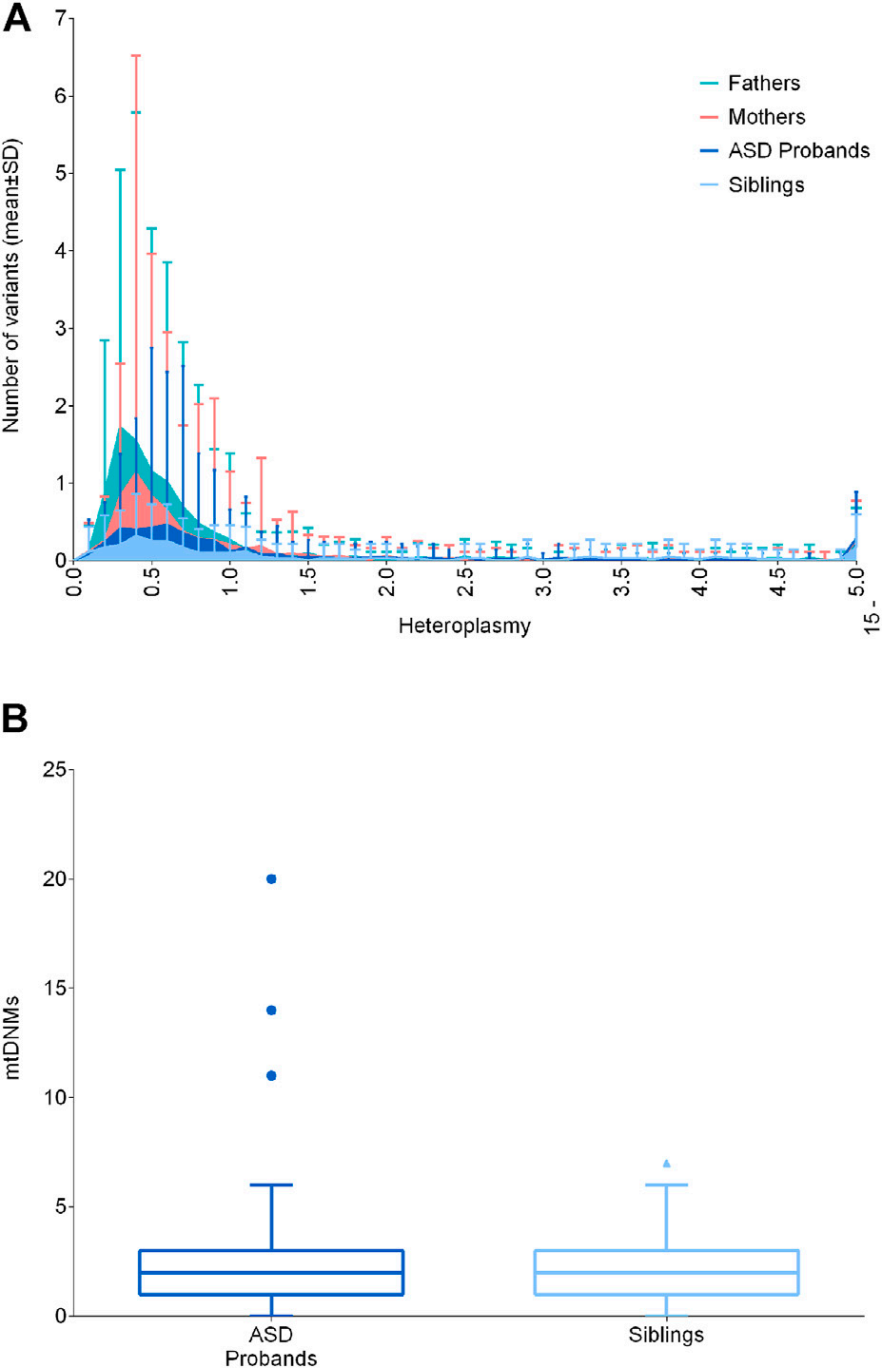
Distribution of low-level heteroplasmic and mtDNA *de novo* variants. **(A)** Frequency of low-level heteroplasmic variants below 15% of heteroplasmy in ASD probands, siblings, mothers and fathers. Heteroplasmy was distributed in classes increasing of 0.1% until 4.9%, and a single class from 5% to 15%. Data are shown as mean ± standard deviation (SD) **(B)** *De novo* variants in ASD probands and siblings, comparing the variants between 0.2% and 15% of heteroplasmy not inherited from mothers. Data are shown as Tukey box and whiskers plot.

Cumulatively, low-level heteroplasmic variants may have biological effects, possibly contributing to pathological manifestations. Such variants may be somatic or maternally inherited and their heteroplasmic load may vary from mother to offspring, defining the so-called “universal heteroplasmy” (Stewart and Chinnery, 2021). Since the mitogenome sequencing in the test cohort was carried out by NGS reaching a high coverage (mean coverage of 16.718X ± 4.617 SD, Supplementary Figure S1), we were able to detect variants with extremely low levels of heteroplasmy (<1%). Thus, we focused on variants with heteroplasmy up to 15%, capturing all other variants not included in the previous analyses. For this analysis, we excluded one sample (BEL39.1) belonging to the “fathers” group, since it presented a 10.7% of contamination, as assessed by Haplocheck. We stratified the number of variants in classes of heteroplasmy and compared ASD probands with their unaffected siblings, mothers and fathers, failing to detect any difference (Figure 2A). Moreover, our results showed that variants below 1% were the most frequent, and their frequencies increased in fathers and mothers, possibly due to the age-related accumulation of somatic mutations (Szczepanowska and Trifunovic, 2017). We also assessed the number of putative mtDNA *de novo* variants, thus comparing the variants between 0.2% and 15% of heteroplasmy not inherited from mothers, according to the tissue investigated (blood cells), failing to detect differences between ASD patients and unaffected siblings (Figure 2B). We detected 438 *de novo* heteroplasmic variants from 176 mother–offspring pairs, specifically 295 *de novo* heteroplasmic variants from 117 mother–ASD offspring pairs and 143 *de novo* heteroplasmic variants from 59 mother–unaffected offspring pairs. The mtDNA mutational rate, calculated as N *de novo*/(N mother–offspring pairs × 16,569 mtDNA bp), was similar in ASD patients (1.52 × 10^−4^) and in unaffected siblings (1.46 × 10^−4^). To compare the mtDNA mutational rate with literature data (Rebolledo-Jaramillo et al., 2014; Wei et al., 2019), we considered all variants over 1% (1%–100%), detecting 162 *de novo* heteroplasmic variants from 176 mother–offspring pairs, specifically 104 *de novo* heteroplasmic variants from 117 mother–ASD offspring pairs and 58 *de novo* heteroplasmic variants from 59 mother–unaffected offspring pairs. Thus, the mtDNA mutational rate was again similar in ASD patients (5.36 × 10^−5^) and in unaffected siblings (5.93 × 10^−5^). Wei and others estimated a mtDNA mutation rate of 1.18 × 10^−5^, using 477 *de novo* heteroplasmic variants from 1,526 mother–offspring pairs, obtained by whole-genome sequencing (WGS). Rebolledo-Jaramillo and others estimated a mtDNA mutation rate of 7.9 × 10^−5^, using 51 *de novo* heteroplasmic variants from 39 mother–offspring pairs, obtained by mtDNA sequencing, starting from two long range amplicons. Both studies showed the same order of magnitude of our data (10^−5^), and slight differences may be accounted to different sequencing approaches and initial mtDNA amplification (Rebolledo-Jaramillo et al., 2014; Wei et al., 2019).

In cells, mtDNA is present in multiple copies and in variable content, depending on the specific energetic requirements or on particular physiological or pathological conditions. Increased mtDNA levels are often indicative of activated mitochondrial biogenesis as compensatory response to sustain ATP production, whereas mtDNA depletion is usually associated with specific mitochondrial diseases caused by defective mtDNA replication or maintenance (Carelli and Chan, 2014). Since previous studies reported both elevated and reduced mtDNA content in ASD patients (Chen et al., 2015; Valiente-Pallejà et al., 2018; Singh et al., 2020), we also quantified mtDNA content from blood cells in the test cohort.

**FIGURE 3:**
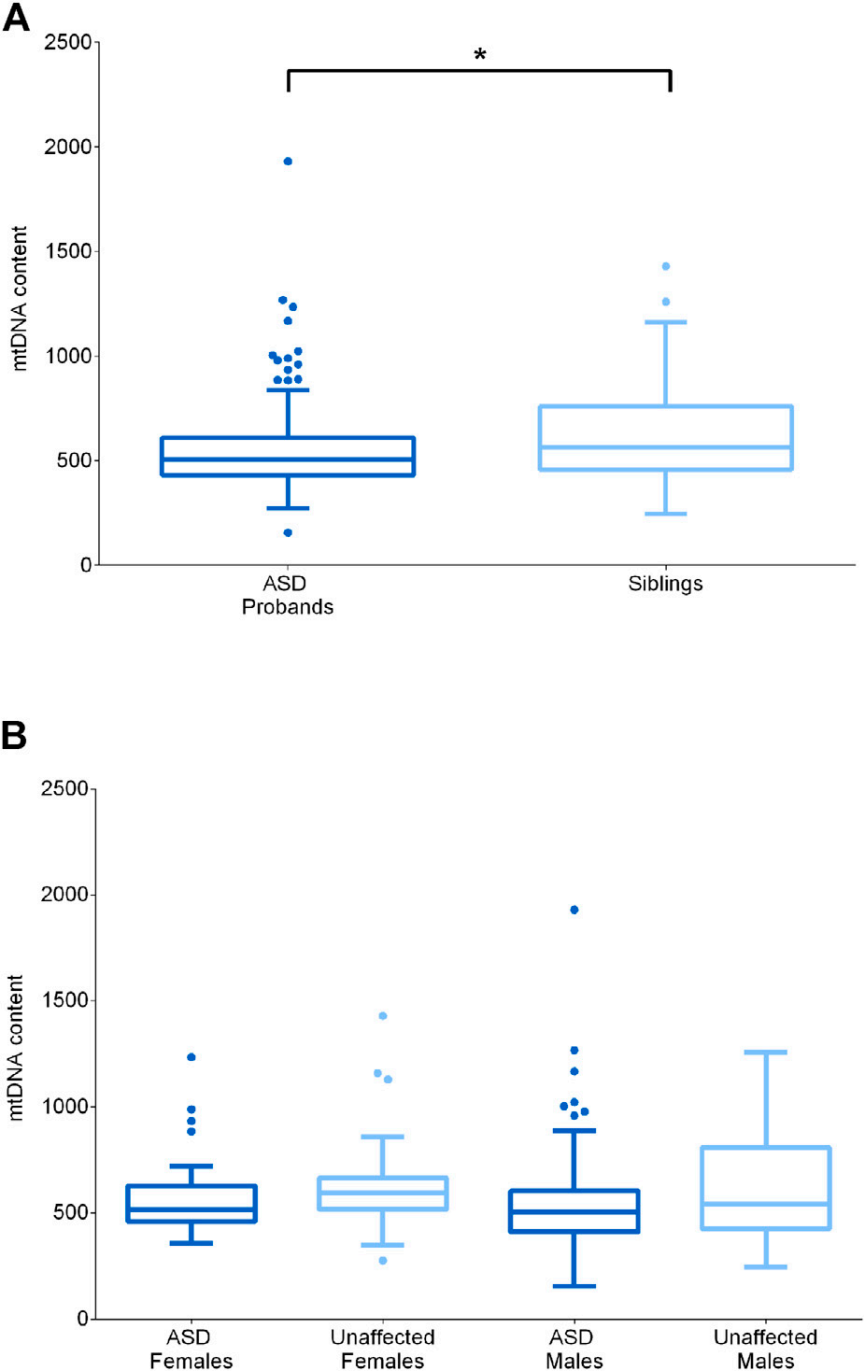
MtDNA content assessment in ASD probands and their unaffected siblings. **(A)** MtDNA content in ASD probands (n = 109, median = 505) and their unaffected siblings (n = 51, median = 564). **(B)** MtDNA content in unaffected females (n = 24, median = 595), ASD females (n = 23, median = 514), unaffected males (n = 27, median = 542) and ASD males (n = 86, median = 504). Data are shown as Tukey box and whiskers plot. * indicates statistical significance (*p*-value <0.05), as calculated by the Mann-Whitney *U*-test.

It is known from literature that mtDNA levels are influenced by gender, probably due to estrogens, and by age (Filograna et al., 2021). To exclude possible effects of age and sex, we compared ASD subjects with their healthy siblings, which were comparable for age, and, in addition, we considered separately males and females. We first compared the whole ASD (n = 109) and sibling (n = 51) groups, highlighting a significantly reduced mtDNA content in the ASD subjects (*p*-value *=* 0.0339) (Figure 3A). Stratifying by gender (F unaffected n = 23; F ASD n = 24; M unaffected n = 27; M ASD n = 86) did not emerge a specific gender impact and significance was lost, possibly due to the reduction in numbers for each individual category (Figure 3B).

### Multivariable regression analyses for ASD susceptibility and severity

To evaluate if a combination of multiple risk factors may act on ASD susceptibility and the severity of the clinical phenotype, we used multivariable regression analyses with a stepwise approach.

**FIGURE 4:**
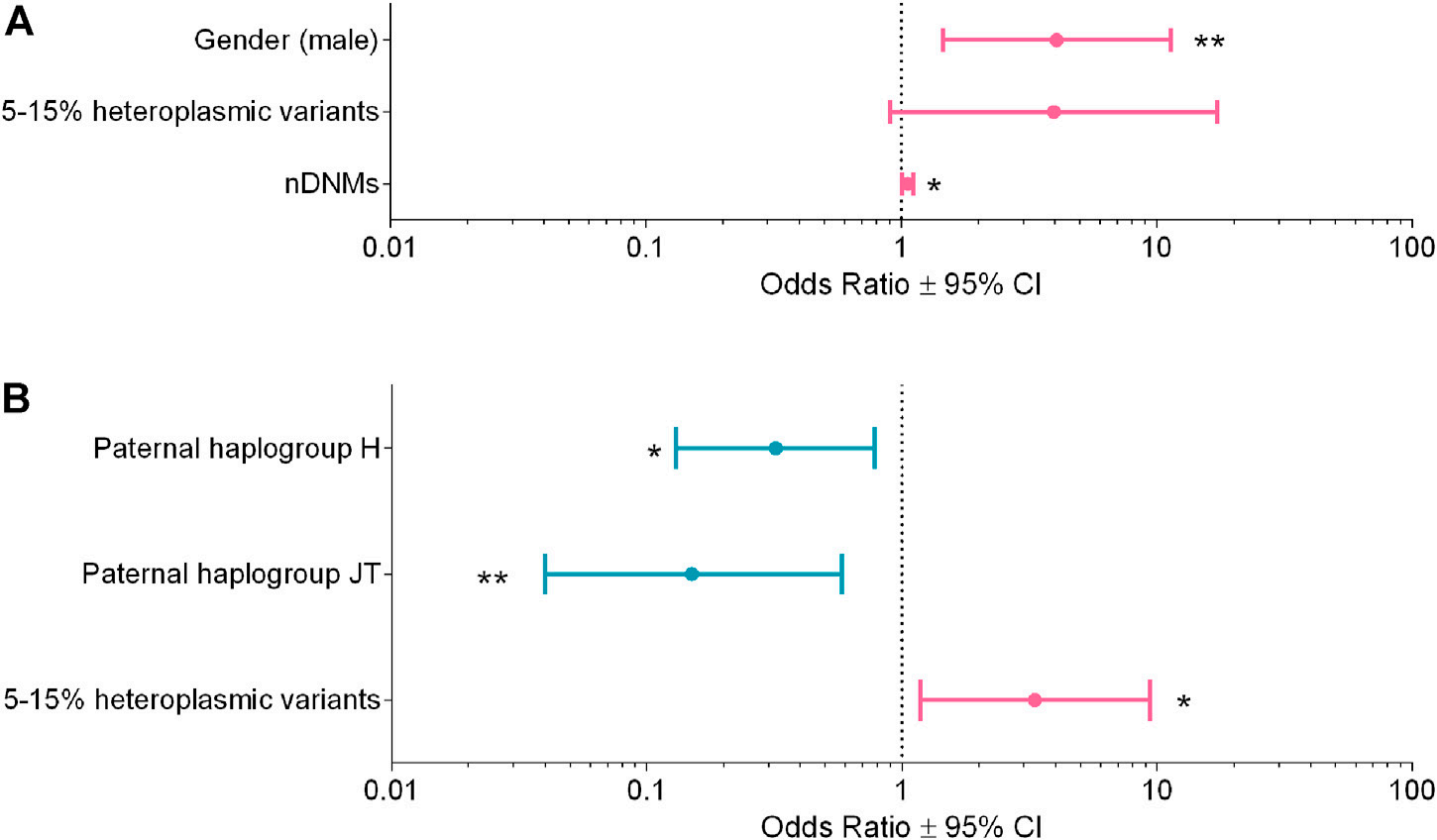
Multivariable logistic regression analyses for ASD susceptibility and severity. **(A)** Analysis for ASD susceptibility, *R* ^2^ = 0.17. **(B)** Analysis for ASD severity *R* ^2^ = 0.14. Odds ratios (OR ± 95% CI) calculated with the multivariable logistic regression are shown in the figure; the variables reducing risk are in blue, whereas those increasing risk are in magenta. * *p*-value <0.05, ** *p*-value <0.01.

In the first regression analysis, we selected only families with at least one unaffected sibling, thus comparing n = 60 ASD subjects vs*.* n = 59 unaffected siblings (Supplementary Table S5). The final model showed the following variables associated with the risk of ASD: gender (male vs*.* female), OR = 4.05, 95% CI = 1.45–11.30, *p*-value = 0.007; nuclear DNMs, OR = 1.06, 95% CI = 1.01–1.11, *p*-value = 0.022; 15%–5% mtDNA variants, OR = 3.95, 95% CI = 0.90–17.20, *p*-value = 0.067 (Figure 4A), with an *R* ^2^ = 0.17.

In the second regression analysis, we compared n = 52 “mild/moderate” vs*.* n = 65 “severe” phenotype, based on the ADOS-2 criteria (Supplementary Table S6). The final model showed the following variables associated to the severity of the clinical phenotype: paternal super-haplogroups H (OR = 0.32, 95% CI = 0.13–0.78, *p*-value = 0.013) and JT (OR = 0.15, 95% CI = 0.04–0.58, *p*-value = 0.006), both associated to milder phenotypes, whereas mtDNA variants with 15%–5% heteroplasmy (OR = 3.32, 95% CI = 1.18–9.35, *p*-value = 0.023) were associated to severe phenotypes (Figure 4B). The *R* ^2^ of this model was 0.14.

## Discussion

In the current study, we comprehensively assessed the mitogenome of blood cells of a collection of 98 ASD families in order to explore the contribution of mtDNA in ASD susceptibility. We investigated all layers of possible mtDNA involvement. First, we studied the mtDNA sequence level, i.e., 1) haplogroups, 2) homoplasmic and heteroplasmic private variants and pathogenic mutations, and 3) low “universal” heteroplasmy. Then, we also assessed the mtDNA amount. We found some signals of mtDNA involvement in ASD pathogenesis and severity, which include, surprisingly, also the paternal mtDNA. Specifically, we highlighted a significant lower abundance of mtDNA copies in ASD probands as compared to their unaffected siblings of comparable age, which was not gender driven. Moreover, evaluating a combination of possible risk factors for ASD, we identified a few that impacted on ASD susceptibility. We confirmed the role played by male gender and the number of nuclear DNMs, with a non-significant tendency suggesting that the presence of mtDNA heteroplasmic variants with loads between 15% and 5% may contribute to the ASD risk. The impact of these variants became significant for increasing the risk of having a severe form of ASD, which was instead reduced by the paternal haplogroups H or JT. Overall, we substantiate a role of mtDNA in the polygenic determination of ASD, confirming some indications, but also highlighting differences from previous reports.

The maternally-inherited mtDNA of ASD probands is the first candidate for a contributory role of the mitogenome. However, our analysis excluded a role for maternal haplogroups in predisposing or protecting from the risk to develop ASD.

Focusing the mitogenome analysis on private missense variants, we found that those affecting the two genes encoding for Complex V subunits were more frequent in the ASD probands, 60% of which were predicted as *possibly* pathogenic. Although not statistically significant after FDR correction, this result is in line with the hypothesis that, on the functional ground, a slight inefficiency of OXPHOS could contribute to ASD pathogenesis, as recently remarked in an animal model of mild Complex I deficiency (Yardeni et al., 2021). To truly validate on the functional ground the impact of these Complex V variants, an appropriate *in vitro* biochemical assessment or study should be performed. From previous literature there is some convergence on both functional (Giulivi et al., 2010) and genetic grounds (Piryaei et al., 2012; Patowary et al., 2017; Valiente-Pallejà et al., 2018), involving mild dysfunction of Complexes I and V. Features resembling ASD, on the other hand, have been occasionally described in well-defined mitochondrial disorders, like in the MELAS syndrome (Pons et al., 2004; Connolly et al., 2010), which is most frequently due to tRNA and Complex I mutations (La Morgia et al., 2020). Our analysis did not highlight a clear role for tRNA variants, as out of the 22 found, only one was predicted as likely pathogenic and other two were heteroplasmic, however not excluding their pathogenic contribution in specific maternal lineages, as occasionally reported (Graf et al., 2000).

The burden of low-level heteroplasmy, defined “universal” heteroplasmy (Payne et al., 2013), was also one of our objects of investigation, as it was recently implicated, for example, in aging and brain development (Ross et al., 2013). Remarkably, our current study provides the deepest mean coverage in NGS sequencing for this type of analysis (16.718X), when compared to the only study previously performed (512X) (Valiente-Pallejà et al., 2018). The vast majority of other studies used various less powered approaches (Citrigno et al., 2020). While we observed a predictable age-related increase of variants with heteroplasmic load <1%, distinguishing parents from offspring, we failed to highlight any difference in the number of heteroplasmic variants between ASD probands and unaffected siblings, as well as in mtDNA DNMs. However, using a multivariable regression analysis, which considers presence or absence of 15%–5% heteroplasmic variants, their contributory role emerged with about a four-fold increase of risk for severe phenotypes. It must be remarked that blood cells, as DNA source, undergo a fast turnover and, as a consequence, these cells are under selective pressure that eliminates potentially pathogenic deviations of mtDNA quality, as well established for clearly pathogenic mtDNA mutations leading, for example, to MELAS syndrome (Grady et al., 2018). This suggests that for reliable information on the impact of “universal” heteroplasmy a post-mitotic tissue would be better suited, such as skeletal muscle, which however imposes limitations due to the invasiveness of muscle biopsy sampling for research purposes.

In addition to the mtDNA sequence, we also assessed mtDNA copy number. This is a signature of compensatory activation of mitochondrial biogenesis, which may be reactive to mild mitochondrial dysfunction, as well established for mitochondrial diseases (Giordano et al., 2014; Filograna et al., 2021). A significant lower mtDNA copy number was observed in the comparison of ASD probands with their unaffected siblings, which was not driven by gender since male and female ASD probands had comparable mtDNA content. This issue has been previously explored with contrasting results. Two studies reported, similar to our results, a reduced mtDNA copy number in blood cells (Valiente-Pallejà et al., 2018; Singh et al., 2020). A third very recent study performed on a very large number of patients from two cohorts solidly shows results similar to ours, linking the reduction of mtDNA copy number to the impact of potentially pathogenic heteroplasmic variants, ultimately impinging on the severity of clinical phenotype (Wang et al., 2022). On the opposite side, a few other studies documented the increase of mtDNA content in blood cells (Giulivi et al., 2010; Chen et al., 2015). The reason for these discrepancies may be multiple, including characteristics of group comparisons, such as age, sub-phenotypes or gender matching. Methodological issues may also play a role, for example whole blood as compared to peripheral blood mononuclear cells, or method of DNA extraction and conservation of samples, with multiple freezing and thawing (Andreu et al., 2009; Guo et al., 2009; Çalışkan et al., 2014). One noticeable observation, both in our and other studies, is the possible coexistence of subpopulations of patients with activated mitochondrial biogenesis that may variably impact on the average assessment. In fact, in our cohort there is a consistent subgroup of outliers with higher mtDNA amount, similar to what previously noticed in a smaller study (Giulivi et al., 2010). It is also known that mtDNA copy number regulation is affected by many factors, both genetic and environmental, as for example physical activity in skeletal muscle (Clay Montier, 2009; Filograna et al., 2021). This issue may be solved by a properly designed larger study, with an appropriate methodology and recruitment of study cohort.

Besides the thorough analysis of maternal mtDNA, the most unpredicted result of our study came from the fathers’ mtDNA analyses. The initial association of paternal mtDNA haplogroups (U5a) in the test cohort was not replicated in a second cohort. This initial association might be explained by a distortion of paternal age at conception in the first cohort. In fact, an elevated fathers’ age at conception is related with a higher accumulation of nuclear DNMs in the offspring, which is a well-known risk factor for ASD, as clearly evinced from the previous literature (Goldmann et al., 2019). In our test cohort we had the possibility to relate paternal haplogroups with these two variables, finding that fathers carrying haplogroups U5a and K were, on average, older at conception than the other fathers and their offspring had the higher number of DNMs. However, a second indication of paternal mtDNA impact came from the multivariate modeling, which highlighted paternal super-haplogroups H and JT as associated with milder ASD phenotypes.

On a speculative ground, multiple hypothesis may be envisioned to explain the observed impact of paternal mtDNA on the pathogenesis of ASD. First, specific mtDNA haplogroups may be prone to generate different rates of nuclear DNMs in individual sperms, considering this mechanism adjunctive to fathers’ age at conception (Kong et al., 2012). A second conceivable way for paternal mtDNA to impact on sperm genome is the possible differential epigenetic profile induced by different mtDNA haplogroups, as suggested by some studies (Bellizzi et al., 2012; Atilano et al., 2015; Lopes, 2020). The epigenetic profile in early phases of development is already well established as influential to ASD pathogenesis (Feinberg et al., 2015; Masini et al., 2020; Garrido et al., 2021). Furthermore, as our analysis did not take into consideration the nuclear genome variability, we cannot exclude that specific mtDNAs may drag specific nuclear DNA variant configurations as the two genomes tightly interact, as recently documented (Wei et al., 2019). In any case we believe this is the first instance in which the paternal mitogenome emerges as impacting on the fate of offspring, contributing to the pathogenic determination of a disease such as ASD. Our current findings only raised the possibility of this scenario, which needs appropriate larger confirmatory investigations.

There are a few limitations intrinsic to our study. First, the sample size would be desirable to be larger than the one gathered from a single center. Second, we have been able to use a second cohort to replicate our initial results only for the haplogroup analysis, as we did not have available neither the DNA nor the clinical data. Thus, some of our results must be considered preliminary and prompt further investigations. Finally, there is an issue exquisitely linked to the source of mtDNA, for which, as we already mentioned, a post-mitotic tissue might be much more informative, as blood cells undergo selective pressure due to their fast turnover. On the positive side, this is among the few systematic mtDNA survey with deep sequencing using all components of ASD families, including both parents and healthy siblings. Furthermore, the paternal haplogroup analysis is attempted here for the first time in relation to the severity of the clinical phenotype in the offspring.

Finally, two observations inferred by our study are worth for the field of mitochondrial biology and medicine. First, we did not detect any trace of mtDNA paternal inheritance, reinforcing the evidence that mitogenomes are strictly maternally inherited, despite recurrent controversies on this issue (Schwartz and Vissing, 2002; Luo et al., 2018; Wei et al., 2020; Bai et al., 2021). Second, the incidental identification in one sibling (brother of a probands) of a clearly pathogenic mutation (m.3890G>A/*MT-ND1*) over 176 mother-child segregations from 98 mothers fits the previous figure of one mtDNA pathogenic mutation carrier every 200 individuals (Elliott et al., 2008). Due to the low heteroplasmy, this mutation found in a male most probably will remain a lifelong silent molecular anomaly, contributing to the large reservoir of mtDNA pathogenic mutations present in the general population (Stewart and Chinnery, 2021).

In conclusion, we contributed to enlarge the landscape of how mtDNA may influence ASD risk, pathogenesis and phenotypic expression. Strikingly, we introduced an unexpected variable, which is the paternal mtDNA, a topic that needs specific experimental design to be confirmed and well understood.

## Data Availability

The original contributions presented in the study are publicly available. This data can be found here: NCBI, PRJNA858216. Whole Genome sequences are deposited at the Database of Genotypes and Phenotypes (dbGaP) (NCBI) with accession number phs002509.v1.p1.
