# Supplementary material for: Dissecting the multifaceted contribution of the mitochondrial genome to autism spectrum disorder
Source: Front Genet. 2022 Nov 7;13:953762. doi: 10.3389/fgene.2022.953762 (PMC9676943; doi:10.3389/fgene.2022.953762)
Supplement: Supplementary file 4 [file DataSheet1.docx]

**Supplemental information**

**Dissecting the multifaceted contribution of the mitochondrial genome to autism spectrum disorder.**

Leonardo Caporali,^1^ Claudio Fiorini,^1^ Flavia Palombo,^1^ Martina Romagnoli,^1^ Flavia Baccari,^2^ Corrado Zenesini,^2^ Paola Visconti,^3^ Annio Posar,^3,4^ Maria Cristina Scaduto,^3^ Danara Ormanbekova,^1^ Agatino Battaglia,^5^ Raffaella Tancredi,^5^ Cinzia Cameli,^6^ Marta Viggiano,^6^ Anna Olivieri,^7^ Antonio Torroni,^7^ Elena Maestrini,^6^ Magali Jane Rochat,^8^ Elena Bacchelli,^6^ Valerio Carelli,^1,4*^ Alessandra Maresca^1*^

^1^IRCCS Istituto delle Scienze Neurologiche di Bologna, Programma di Neurogenetica, Bologna, 40139, Italy.

^2^IRCCS Istituto delle Scienze Neurologiche di Bologna, UOSI Epidemiologia e Statistica, Bologna, 40139, Italy.

^3^IRCCS Istituto delle Scienze Neurologiche di Bologna, UOSI Disturbi dello Spettro Autistico, Bologna, 40139, Italy.

^4^Department of Biomedical and Neuromotor Sciences, University of Bologna, 40139, Italy.

^5^IRCCS Stella Maris Foundation, Department of Developmental Neuroscience, Pisa, 56128, Italy.

^6^Department of Pharmacy and Biotechnology, University of Bologna, 40126, Italy.

^7^Department of Biology and Biotechnology "L. Spallanzani", University of Pavia, Pavia, 27100, Italy.

^8^IRCCS Istituto delle Scienze Neurologiche di Bologna, Programma Diagnostica Funzionale Neuroradiologica, Bologna, 40139, Italy.

^*^Correspondence: Valerio Carelli, MD, PhD, valerio.carelli@unibo.it

^**^Correspondence: Alessandra Maresca, PhD, alessandra.maresca@isnb.it

**Description of supplemental contents**

**Table S1 (file Table S1.xlsx) -** Database of the test cohort reporting haplogroups assignment and private variants.

**Table S2 (file Table S2.xlsx) –** Database of the test cohort reporting missense variants (private variants) in coding genes for each subjects with related pathogenicity classification.

**Table S3 (file Table S3.xlsx) -** Database of the test cohort reporting private variants in tRNA genes for each subjects with related pathogenicity classification

**Table S4 -** Private missense variants distribution in genes and OXPHOS complexes in the Italian ASD maternal lines from the test cohort, compared to Italian healthy controls.

**Figure S1 -** Coverage throughout the entire mitochondrial genome.

**Table S5 -** Descriptive statistics (left) and outputs of the univariate conditional logistic regression (right) for the ASD susceptibility analysis.

**Table S6**- Descriptive statistics (left) and outputs of the univariate generalized mixed linear regression (right) for the ASD severity analysis.

**Table S4. Private missense variants distribution in genes and OXPHOS complexes in the Italian ASD maternal lines from the test cohort, compared to Italian healthy controls.**

|  |  | **Maternal** | | | | **CTRLs** | |
| --- | --- | --- | --- | --- | --- | --- | --- |
| **Complex** | **Gene** | **N** | **%** | **p-value** | **q-value** | **N** | **%** |
| I |  | 11 | 14.9 | 0.52 | 0.52 | 37 | 15.3 |
|  | *MT-ND1* | 1 | 1.4 | 1.00 | 1.00 | 7 | 2.9 |
|  | *MT-ND2* | 0 | 0.0 | 0.31 | 0.90 | 6 | 2.5 |
|  | *MT-ND3* | 1 | 1.4 | 1.00 | 1.00 | 3 | 1.2 |
|  | *MT-ND4* | 0 | 0.0 | 0.12 | 0.80 | 4 | 1.7 |
|  | *MT-ND4L* | 1 | 1.4 | 0.55 | 0.90 | 1 | 0.4 |
|  | *MT-ND5* | 7 | 9.5 | 0.36 | 0.90 | 11 | 4.6 |
|  | *MT-ND6* | 1 | 1.4 | 1.00 | 1.00 | 5 | 2.1 |
| III |  | 1 | 1.4 | 0.22 | 0.44 | 17 | 7.0 |
|  | *MT-CYB* | 1 | 1.4 | 0.22 | 0.90 | 17 | 7.0 |
| IV |  | 7 | 9.5 | 0.38 | 0.51 | 20 | 8.3 |
|  | *MT-CO1* | 3 | 4.1 | 0.72 | 1.00 | 6 | 2.5 |
|  | *MT-CO2* | 3 | 4.1 | 0.44 | 0.90 | 6 | 2.5 |
|  | *MT-CO3* | 1 | 1.4 | 0.51 | 0.90 | 8 | 3.3 |
| V |  | 14 | 18.9 | 0.04 | 0.15 | 23 | 9.5 |
|  | *MT-ATP6* | 14 | 18.9 | 0.02 | 0.22 | 20 | 8.3 |
|  | *MT-ATP8* | 0 | 0.0 | 1.00 | 1.00 | 3 | 1.2 |
| No variant | | 41 | 55.4 | - | - | 145 | 59.9 |
| total | | 74 |  |  |  | 242 |  |

Frequencies of missense variants in mtDNA stratified for OXPHOS complexes or genes in the Italian ASD maternal lines (n=74) from the test cohort, compared to Italian healthy controls (n=242). No significant association was found after FDR correction.

**Figure S1**

**
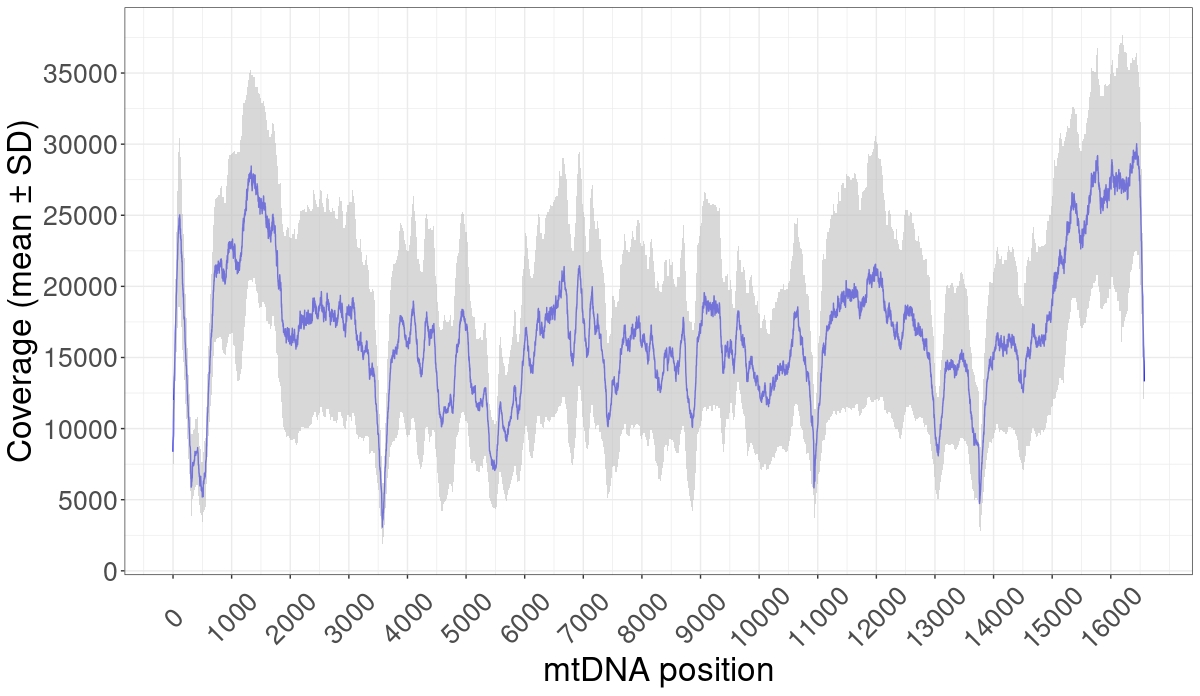
**

**Figure S1. Coverage throughout the entire mitochondrial genome**

Distribution of mean coverage (in purple) with standard deviation (in grey) for all mitogenome nucleotide positions in all samples of study cohort (n=369).

**Table S5- Descriptive statistics (left) and outputs of the univariate conditional logistic regression (right) for the ASD susceptibility analysis.**

Comparisons between the two groups of interest (left) were carried out by Fisher’s exact test or chi-square for categorical data and by Wilcoxon-Mann-Whitney test or t-test for continuous data. SD, standard deviation; OR, odds ratio; CI, confidence interval.

|  | Unaffected siblings  (N=59) | ASD  (N=60) | p-value | OR | 95% CI | p-value |
| --- | --- | --- | --- | --- | --- | --- |
| Maternal age at time of conception (±SD)* | 31.0 ± 5.0 | 32.4 ± 4.9 | 0.146 | 1.14 | 1.02-1.28 | **0.022** |
| Paternal age at time of  conception (±SD)** | 34.1 ± 5.6 | 35.8 ± 5.5 | 0.118 | 1.15 | 1.03-1.29 | **0.016** |
| Gender (N/%)  MALE | 34 / 57.6% | 47 / 78.3% | 0.019 | 2.55 | 1.11-5.88 | 0.028 |
| mtDNA content (±SD)*** | 629.3 ± 254.6 | 613.4 ± 301.0 | 0.373 | 1.00 | 0.99-1.00 | 0.499 |
| mtDNA DNMs (±SD) | 2.5 ± 1.6 | 2.5 ± 2.7 | 0.326 | 1.02 | 0.86-1.21 | 0.833 |
| Subjects with variants at 15-5% heteroplasmy (N/%) | 8 / 13.6% | 12 / 20.0% | 0.463 | 2.63 | 0. 68-10.12 | 0.163 |
| Subjects with variants at 4.9-1% heteroplasmy (N/%) | 39 / 66.1% | 41 / 68.3% | 0.847 | 1.10 | 0.46-2.62 | 0.825 |
| Subjects with variants at 0.9-0.5% heteroplasmy (N/%) | 34 / 57.6% | 41 / 68.3% | 0.258 | 1.62 | 0.71-3.69 | 0.248 |
| Subjects with variants at 0.4-0% heteroplasmy (N/%) | 32 / 54.2% | 27 / 45.0% | 0.361 | 0.66 | 0.29-1.52 | 0.330 |
| Nuclear DNMs (±SD)**** | 78 .4 ± 13.9 | 79.8 ± 16.6 | 0.727 | 1.03 | 0.99-1.07 | 0.181 |

*1 missing in the unaffected siblings group.

**5 missing, 3 in the unaffected siblings group and 2 in the ASD group.

***12 missing, 6 in the unaffected siblings group and 6 in the ASD group.

****10 missing, 5 in the unaffected siblings group and 5 in the ASD group.

**Table S6**- **Descriptive statistics (left) and outputs of the univariate generalized mixed linear regression (right) for the ASD severity analysis.**

Comparisons between the two groups of interest (left) were carried out by Fisher’s exact test or chi-square for categorical data and by Wilcoxon-Mann-Whitney test or t-test for continuous data. SD, standard deviation; OR, odds ratio; CI, confidence interval; NC, not calculated.

|  | Low-mild  (N=52/44.4%) | Severe  (N=65/55.6%) | p-value | Total  (N=117) | OR | 95% CI | p-value |
| --- | --- | --- | --- | --- | --- | --- | --- |
| Maternal age at  time of conception (SD) | 31.9 (4.4) | 32.86 (4.8) | 0.260 | 32.42 (4.6) | 1.09 | 0.92-1.30 | 0.300 |
| Paternal age at  time of conception (SD)* | 35.6 (5.4) | 37.47 (5.8) | 0.083 | 36.66 (5.7) | 1.13 | 0.96-1.33 | 0.133 |
| Gender (%)  M | 42 (80.8) | 51 (78.5) | 0.821 | 93 (79.5) | 1.35 | 0.22-8.24 | 0.746 |
| Family type (%)  SPX | 30 (57.7) | 48 (73.9) | 0.066 | 78 (66.7) | 3.20 | 0.57-17.88 | 0.185 |
| mtDNA content (SD)** | 593.12 (255.0) | 551.96 (232.8) | 0.586 | 570.25 (242.7) | 1.00 | 0.99-1.00 | 0.397 |
| Super Haplogroup (%)  H  JT  UK  OTHERS | 18 (34.6)  6 (11.5)  8 (15.4)  20 (38.5) | 20 (30.8)  8 (12.3)  11 (16.9)  26 (40.0) | 0.659  0.899  0.823  0.866 | 38 (32.5)  14 (12.0)  19 (16.1)  46 (39.4) | 0.90  0.98  1.04  1.09 | 0.19-4.20  0.11-8.3  0.16-6.92  0.24-4.90 | 0.890  0.983  0.962  0.911 |
| Haplogroup (%)  H*  H1  I  J  K  L  T  U  Others | 11 (21.2)  7 (13.5)  1 (1.9)  2 (3.9)  2 (3.9)  2 (3.9)  4 (7.7)  6 (11.5)  17 (32.7) | 16 (24.6)  4 (6.2)  4 (6.2)  2 (3.1)  2 (3.1)  3 (4.6)  6 (9.2)  9 (13.9)  19 (29.2) | 0.659  0.213  0.380  0.999  0.999  0.999  0.999  0.711  0.687 | 27 (23.1)  11 (9.4)  5 (4.3)  4 (3.4)  4 (3.4)  5 (4.3)  10 (8.6)  15 (12.8)  36 (30.8) | 2.02  0.20  5.59  0.55  0.55  0.79  1.25  1.26  0.84 | 0.31-13.20  0.01-2.94  0.12-257.28  0.01-25.96  0.01-25.96  0.02-31.16  0.10-14.92  0.16-10.11  0.17-4.21 | 0.460  0.240  0.378  0.764  0.764  0.898  0.861  0.826  0.835 |
| Paternal Super Haplogroup (%)**  H  JT  UK  OTHERS | 20 (39.3)  11 (21.6)  12 (23.5)  8 (15.7) | 14 (24.6)  4 (7.0)  24 (42.1)  15 (26.3) | 0.045  0.024  0.107  0.354 | 34 (31.5)  15 (13.9)  36 (33.3)  23 (21.3) | 0.24  0.06  2.96  1.75 | 0.04-1.44  0.01-1.31  0.56-15.64  0.30-10.07 | 0.120  0.074  0.202  0.340 |
| Paternal Haplogroup (%)**  H  J  K  T  U*  U5a  Others | 20 (39.3)  6 (11.8)  3 (5.8)  5 (9.6)  6 (11.5)  3 (5.8)  8 (15.7) | 14 (24.6)  2 (3.5)  9 (13.9)  2 (3.1)  8 (12.3)  7 (10.8)  15 (26.3) | 0.045  0.136  0.222  0.239  0.899  0.509  0.354 | 34 (29.1)  8 (7.4)  12 (10.3)  7 (6.0)  14 (12.0)  10 (8.6)  23 (21.3) | 0.24  0.07  3.69  0.08  0.99  5.51  1.75 | 0.04-1.44  0.00-2.33  0.32-42.46  0.00-4.34  0.11-8.58  0.27-114.08  0.30-10.07 | 0.120  0.137  0.295  0.213  0.994  0.269  0.340 |
| Subjects with ≥1 potential pathogenic mutations (%) | 5 (9.6) | 15 (23.1) | 0.055 | 20 (17.1) | 6.21 | 0.64-60.27 | 0.115 |
| Subjects with ≥1 potential pathogenic mutations in CI (%) | 2 (3.8) | 1 (1.5) | 0.584 | 3 (2.6) | 0.30 | 0.00-37.37 | 0.623 |
| Subjects with ≥1 potential pathogenic mutations in CIII (%) | 1 (1.9) | 0 (0.0) | 0.444 | 1 (0.9) | NC | NC | NC |
| Subjects with ≥1 potential pathogenic mutations in CIV (%) | 2 (3.8) | 2 (3.1) | 0.999 | 4 (3.4) | 1.17 | 0.02-79.88 | 0.942 |
| Subjects with ≥1 potential pathogenic mutations in CV (%) | 2 (3.8) | 11 (16.9) | 0.036 | 13 (11.1) | 11.11 | 0.73-169.03 | 0.083 |
| Subjects with ≥1 potential pathogenic mutations in tRNAs (%) | 0 (0.0) | 1 (1.5) | 0.999 | 1 (0.9) | NC | NC | NC |
| Subjects with ≥1 missense mutation in CI (%) | 14 (26.9) | 11 (16.9) | 0.190 | 25 (21.4) | 0.40 | 0.06-2.57 | 0.335 |
| Subjects with ≥1 missense mutation in CIII (%) | 3 (5.8) | 1 (1.5) | 0.322 | 4 (3.4) | 0.07 | 0.00-5.31 | 0.224 |
| Subjects with ≥1 missense mutation in CIV (%) | 8 (15.4) | 8 (12.3) | 0.630 | 16 (13.7) | 0.64 | 0.07-5.56 | 0.683 |
| Subjects with ≥1 missense mutation in CV (%) | 6 (11.5) | 13 (20.0) | 0.218 | 19 (16.2) | 2.34 | 0.33-16.45 | 0.392 |
| Subjects with ≥1 missense mutation in Trna (%) | 13 (25.0) | 12 (18.5) | 0.391 | 25 (21.4) | 0.59 | 0.10-3.41 | 0.553 |
| Subjects with variants at 15-5% heteroplasmy (%) | 7 (13.5) | 21 (32.3) | 0.018 | 28 (23.9) | 3.61 | 0.91-14.34 | 0.068 |
| Subjects with variants at 4.9-1% heteroplamy (%) | 31 (59.6) | 46 (70.8) | 0.206 | 77 (65.8) | 1.45 | 0.38-5.51 | 0.583 |
| Subjects with variants at 0.9-0.5% heteroplasmy (%) | 26 (50.0) | 41 (63.1) | 0.155 | 67 (57.3) | 2.15 | 0.58-7.97 | 0.253 |
| Subjects with variants at 0.4-0% heteroplasmy (%) | 31 (59.6) | 30 (46.2) | 0.148 | 61 (52.1) | 0.48 | 0.13-1.76 | 0.265 |
| mtDNA DNMs >0.2% (SD) | 3.00 (4.1) | 2.69 (2.6) | 0.419 | 2.8 ± 3.3 | 0.95 | 0.76-1.20 | 0.671 |
| Nuclear DNMs (SD)*** | 80.0 (13.1) | 82.2 (16.3) | 0.447 | 81.2 ± 14.9 | 1.01 | 0.97-1.06 | 0.528 |

*3 missing: 2 in the Mild/Moderate group and 1 in the Severe group.

**9 missing: 4 in the Mild/Moderate group and 5 in the Severe group for mtDNA content; 2 in the Mild/Moderate group and 7 in the Severe group for paternal haplogroups.

***8 missing: 2 in the Mild/Moderate group and 6 in the Severe group.
